# Obturator internus pyomyositis manifested as sciatica in a patient with subacute bacterial endocarditis: A rare case report

**DOI:** 10.1097/MD.0000000000004340

**Published:** 2016-07-29

**Authors:** Wei-Ching Hsu, Jin-Yi Hsu, Michael Yu-Chih Chen, Chung-Chao Liang

**Affiliations:** a Department of Physical Medicine and Rehabilitation, Buddhist Tzu Chi General Hospital; b School of Medicine, Tzu Chi University; c Division of Cardiology, Department of Internal Medicine, Buddhist Tzu Chi General Hospital, Hualien, Taiwan.

**Keywords:** endocarditis, obturator internus, pyomyositis, sciatica

## Abstract

Pyomyositis is a pyogenic infection of the skeletal muscles causing myalgia and fever in patients. Hematogenous seeding engendered by persistent bacteremia and septic embolism is usually the underlying cause of the disease. Trauma, intravenous drug use, and immunodeficiency are the main predisposing factors.

Obturator internus pyomyositis with sciatica has not previously been reported. We report a rare case of a patient with subacute bacterial endocarditis presenting with left buttock pain and sciatica.

Computed tomography confirmed the diagnosis of obturator internus pyomyositis. The patient was discharged uneventfully after successful antibiotic treatment.

The mortality rate of patients who have pyomyositis comorbid with another condition or disease is extremely high. Early diagnosis and aggressive management are imperative.

## 1 Introduction

Pyomyositis is an infectious disease of the skeletal muscles and is mostly caused by bacterial infection. It is an infrequent complication of bacterial endocarditis.^[1,2]^ In a prospective case series comprising 11 patients diagnosed with staphylococcal pyomyositis between 1998 and 2007, only 1 patient had bacterial endocarditis. Recent trauma, immunodeficiency, and intravenous drug use are considered to be the main predisposing factors.^[3]^ Most pyomyositis presents with muscle pain and fever. Although some studies have described piriformis pyomyositis manifested as sciatica,^[3–6]^ obturator internus pyomyositis with sciatica has not previously been reported. We herein present a case of obturator internus pyomyositis manifested as sciatica in a patient with subacute bacterial endocarditis. The related literature is also reviewed.

## 2 Ethics statement

The Research Ethics Committee of Hualien Tzu Chi Hospital approved the case report.

## 3 Case report

**Figure 1:**
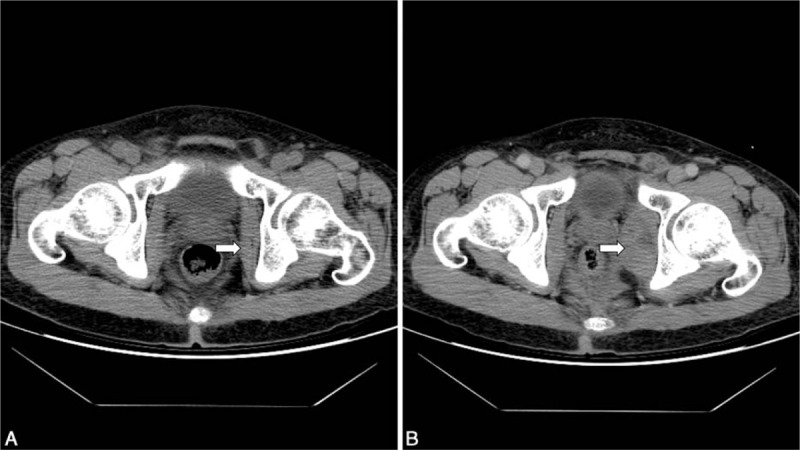
Contrast-enhanced computed tomography (CT) results for the patient: (A) CT conducted at the emergency room, indicating no specific finding of the left obturator internus muscle (arrow); and (B) CT conducted after the patient complained of buttock pain, revealing an enlarged and heterogeneously enhanced soft tissue mass in the left obturator internus muscle (arrow).

A 49-year-old man with a history of chronic liver cirrhosis presented with intermittent fever and chills for 2 days. He had a history of high alcohol intake but had stopped drinking several months earlier. A grade II systolic ejection murmur was detected in the aortic region during auscultation. Transthoracic cardiac echography revealed a small oscillating vegetation at the aortic valve. He was treated empirically with intravenous vancomycin 1000 mg every 12 hours for a diagnosis of subacute bacterial endocarditis. Three pairs of blood cultures were obtained and yielded *Staphylococcus haemolyticus*. Subsequent blood cultures were sterile after 12 days of antibiotic treatment. However, the patient complained of left lower back pain radiating through his buttock to the posterior aspect of the left thigh. A deep tender point over the left gluteal region was found without swelling, warmth, or ecchymosis. A straight leg raise test was administered, but the result was equivocal. The symptoms rapidly deteriorated despite analgesics and the antibiotics. Contrast-enhanced computed tomography (CT) revealed an enlarged left obturator internus muscle with heterogeneous contrast enhancement (Fig. 1). Pyomyositis of the left obturator internus was confirmed and intravenous gentamicin 80 mg every 12 hours was administered. Because no well-liquefied abscess was present, drainage was not considered. The sciatica and myalgia subsided gradually with antibiotics treatment. After a complete course of antibiotics for subacute endocarditis, the patient improved and was discharged uneventfully.

## 4 Discussion

Pyomyositis mostly occurs in tropical areas; however, the incidence in temperate climates has increased in recent years.^[3,4,7–9]^ Its predisposing factors include trauma, intravenous drug use, and immunodeficiency such as HIV infection, diabetes mellitus, malignancy, rheumatologic disorder, liver cirrhosis, and renal insufficiency.^[3]^ Bacteremia is associated with pyomyositis, though the mechanism remains to be elucidated.^[1,10]^ One hypothesized mechanism is initial muscular trauma followed by bacteremia and finally infection of the traumatized muscle.^[3,8]^ A review reported that a third of pyomyositis cases were associated with local trauma or intensive exercise, approximately 30% had a positive blood culture, and approximately 60% involved HIV or another underlying condition.^[3]^

Because pyomyositis is a soft tissue infectious disease, muscle pain and fever are the initial symptoms and are followed by abscess formation and sepsis.^[3,11–13]^ The infection usually affects the extremities and pelvic muscles.^[3,7,11]^ Sciatica cause by pyomyositis of the piriformis or gluteal muscles has been reported in a few patients.^[4–6]^*Staphylococcus aureus* is the most common pathogen; other pathogens have been reported but are rare.^[8]^*S haemolyticus* is one of the coagulase-negative staphylococci, a major nosocomial pathogen, but is rarely reported as a cause of community-acquired native valve infective endocarditis. *S haemolyticus* is mostly isolated from axillae and pubic areas associated with foreign body-related infection and bloodstream infection in neonates.^[14]^ Our patient underwent debridement 3 times and received a split-thickness skin graft operation for a compartment syndrome because of a trauma-related left thigh hematoma 2 months before admission. His bacterial endocarditis and pyomyositis may have been related to this trauma and the following procedure. Magnetic resonance imaging is the first choice for diagnosing pyomyositis because it yields a superior soft tissue contrast compared with CT.^[15]^ The initial management of pyomyositis should be intravenous antibiotics, and drainage should be performed if an abscess forms.^[1,3,15]^

Because the initial presentation of pyomyositis is acute pain in the pelvis or extremities, early diagnosis is sometimes difficult.^[3]^ When the patient has other preexisting medical conditions, the symptoms can be difficult to characterize. In a previous case report, a patient with acute lymphoblastic leukemia was hospitalized for induction chemotherapy and developed pyomyositis that rapidly progressed to quadriparesis and septic shock. The patient presented initially with right hand and left knee pain only.^[8]^ Approximately 80% of patients with pyomyositis, whether immunocompromised or immunocompetent, experience fever.^[3]^ White blood cell count is not a favorable marker for pyomyositis, especially for immunocompromised patients.^[3,16]^ For immunocompetent patients, C-reactive protein (CRP) and erythrocyte sedimentation rate (ESR) may be good laboratory tests for excluding pyomyositis.^[16,17]^ A retrospective review of 199 children with a mean age of 8 years found that no child with a CRP level of less than 3.6 mg/dL or ESR less than 22 mm/h had pyomyositis.^[17]^ For immunocompromised patient, because pyomyositis can progress quickly, early image studies must be performed. Our patient initially reported left buttock pain with sciatica. The symptoms mimicked those of many other diseases and conditions such as simple muscle strain, piriformis syndrome, lumbar spondylosis, and intervertebral disc displacement. The history of liver cirrhosis, bacteremia, and rapidly deteriorated symptoms prompted us to quickly investigate to confirm the diagnosis and prescribe an aggressive antibiotics treatment. The antibiotic regimen was based on empiric therapy for native or prosthetic valve endocarditis according to American Heart Association guideline.^[18]^ Gentamicin was added empirically because infective endocarditis from enterococcal infection is not uncommon in patients with chronic liver disease.

In summary, if a patient with bacteremia complains of local muscular pain with sciatica, the possibility of pyomyositis should be considered, particularly if the patient has predisposing factors. Early diagnosis and prompt medical intervention are crucial in treating such patients.^[3]^
